# Supplementary material for: The Systems Biology Research Tool: evolvable open-source software
Source: BMC Syst Biol. 2008 Jun 29;2:55. doi: 10.1186/1752-0509-2-55 (PMC2446383; doi:10.1186/1752-0509-2-55)
Supplement: Additional file 1 — SBRT Archive. An archive of the current version of the Systems Biology Research Tool. [file 1752-0509-2-55-S1.zip › sbrt-1.4.0/doc/developers_guide/api/sbrt/kernel/fba/proc/package-use.html]

Uses of Package sbrt.kernel.fba.proc


|  |  |  |
| --- | --- | --- |
| **Overview** \| **Package** \| Class \| **Use** \| **Tree** \| **Deprecated** \| **Index** \| **Help** | |  |
| PREV   NEXT | **FRAMES**    **NO FRAMES**     **All Classes** |  |


---


## **Uses of Package sbrt.kernel.fba.proc**

| Packages that use sbrt.kernel.fba.proc | |
| --- | --- |
| **sbrt.kernel.fba.proc** | Provides classes and interfaces for executing Flux Balance Analysis processes. |
| **sbrt.shell.mng.fba** | Provides classes and interfaces for creating Flux Balance Analysis process managers. |

Classes in sbrt.kernel.fba.proc used by sbrt.kernel.fba.proc

****CvFbaOptProcess****
  
          This class is used to represent constraint-variation Flux Balance Analysis
experiments.

****FbaProcess****
  
          This abstract class is used to represent Flux Balance Analysis experiments.

****FluxActivityProcess.Results****
  
          This class is used to represent the results of a flux activity
experiment.

****FluxPlasticityProcess.Results****
  
          This class is used to represent the results of a flux plasticity
experiment.

****NetworkInfoProcess.Results****
  
          This class is used to represent the information gathered about a
fluxome.

Classes in sbrt.kernel.fba.proc used by sbrt.shell.mng.fba

****CatalystDeletionProcess****
  
          This class is used to perform catalyst deletion experiments.

****CdHarFluxVectorProcess****
  
          This class is used to perform coordinate direction hit-and-run flux vector
experiments.

****CvCatalystDeletionProcess****
  
          This class is used to perform constraint-variation catalyst deletion
experiments.

****CvFbaOptimizationExperiment****
  
          This class is used to perform constraint-variation FBA optimization
experiments.

****CvFluxIntervalProcess****
  
          This class is used to perform constraint variation flux interval experiments.

****CvObjectiveFunctionProcess****
  
          This class is used to perform constraint-variation objective function
experiments.

****CvRxnDeletionProcess****
  
          This class is used to perform constraint-variation reaction deletion
experiments.

****CycleIdProcess****
  
          This class is used to execute steady state cycle identification processes.

****ExtremeCurrentProcess****
  
          This class is used to perform extreme current experiments.

****FbaOptimizationProcess****
  
          This class is used to perform basic Flux Balance Analysis optimization.

****FbaProcess****
  
          This abstract class is used to represent Flux Balance Analysis experiments.

****FbaSolverProcess****
  
          This class is used to solve Sv = 0.

****FluxActivityProcess****
  
          This class is used to perform flux activity experiments.

****FluxCapProcess****
  
          This class is used to perform a flux cap identification.

****FluxIntervalProcess****
  
          This class is used to perform flux interval experiments.

****FluxomeReducerProcess****
  
          This class is used to execute fluxome reduction processes.

****FluxPlasticityProcess****
  
          This class is used to perform flux plasticity experiments.

****InitialPointGenerator****
  
          This class is used to perform initial point experiments.

****NetworkInfoProcess****
  
          This class is used to gather basic information about a fluxome.

****ObjectiveFunctionAnalysis****
  
          This class is used to perform objective function experiments.

****RandomConstraintsGeneratorV1****
  
          This class is used to generate random flux constraints.

****RandomEnvGeneratorV1****
  
          This class is used to generate random environments for use in flux balance
analysis.

****RandomObjectiveGenerator****
  
          This class is used to generate random objective functions.

****RxnDeletionProcess****
  
          This class is used to perform reaction deletion experiments.

****StoichVariationProcess****
  
          This class is used to perform stoichiometry-variation FBA optimizations.

****SvCdHarFluxVectorProcess****
  
          This class is used to perform space variation coordinate direction
hit-and-run flux vector experiments.

****SvInitialPointProcess****
  
          This class is used to perform constraint variation initial point experiments.

---


|  |  |  |
| --- | --- | --- |
| **Overview** \| **Package** \| Class \| **Use** \| **Tree** \| **Deprecated** \| **Index** \| **Help** | |  |
| PREV   NEXT | **FRAMES**    **NO FRAMES**     **All Classes** |  |


---
